# Supplementary material for: Targeting OPA1-Mediated Mitochondrial Fusion Contributed to Celastrol’s Anti-Tumor Angiogenesis Effect
Source: Pharmaceutics. 2022 Dec 23;15(1):48. doi: 10.3390/pharmaceutics15010048 (PMC9866574; doi:10.3390/pharmaceutics15010048)
Supplement: Supplementary file 1 [file pharmaceutics-15-00048-s001.zip › pharmaceutics-2031133-supplementary.pdf]

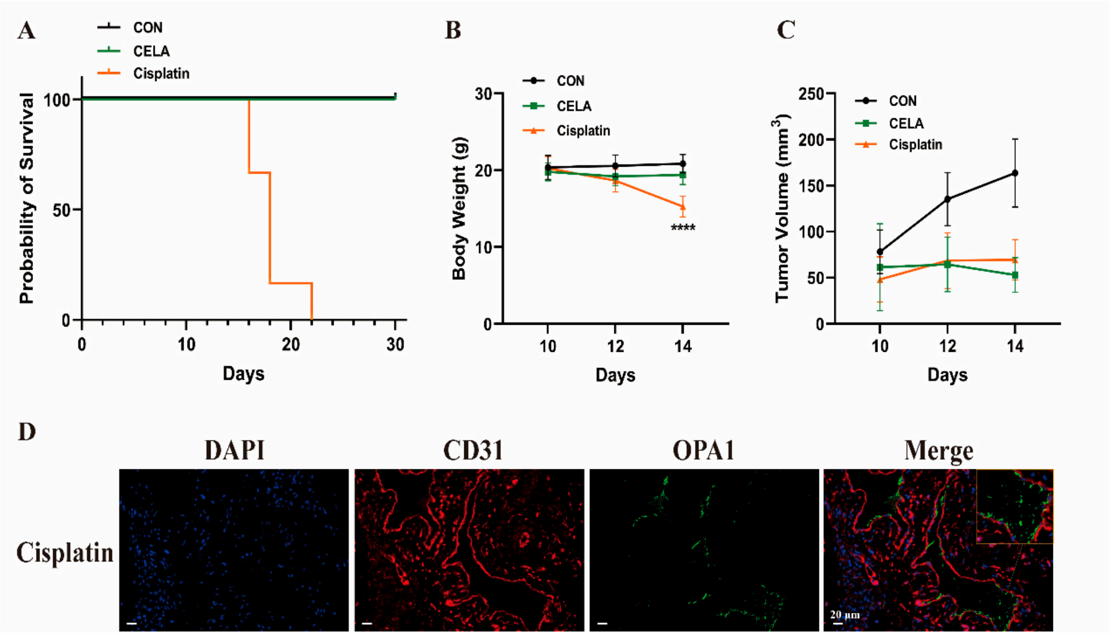

Figure S1. Cisplatin administration did not show a significant inhibitory effect on tumor angiogenesis *in vivo*. (**a**) The survival curve, (**b**) changes in body weight and (**c**) changes in tumor volume under celastrol or cisplatin treatment; (**d**) The expression of CD31 and OPA1 in tumor tissues after 12 day's cisplatin administration. ****$p < 0.0001$.

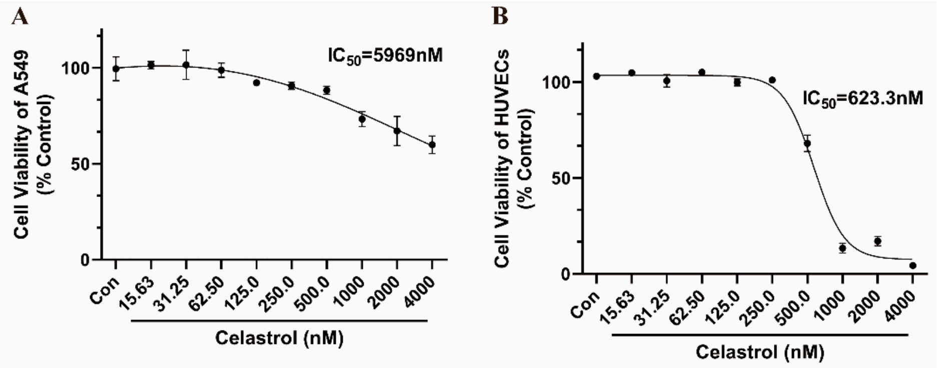

Figure S2. (**a**), (**b**) Cell viability of A549 and HUVECs after 24 h celastrol treatment.
